# Cancer-secreted exosomal miR-21-5p induces angiogenesis and vascular permeability by targeting KRIT1

**DOI:** 10.1038/s41419-021-03803-8

**Published:** 2021-06-04

**Authors:** Qinglian He, Aihua Ye, Weibiao Ye, Xiaomin Liao, Guoqiang Qin, Yongqiang Xu, Yuting Yin, Huanqian Luo, Muhua Yi, Liying Xian, Shihao Zhang, Xiyuan Qin, Wei Zhu, Yuling Li

**Affiliations:** 1 Department of Pathology, Dongguan People’s Hospital affiliated to Southern Medical University, Dongguan, China; 2 Department of Pathology, Guangdong Medical University, Dongguan, China; 3 Department of Obstetrics, Longhua District Maternity and Child Healthcare Hospital, Shenzhen, China; 4 Department of science and education, Dongguan People’s Hospital affiliated to Southern Medical University, Dongguan, China; 5 Department of Ultrasound, Dongguan People’s Hospital affiliated to Southern Medical University, Dongguan, China

**Keywords:** Cancer microenvironment, Colon cancer

## Abstract

Cancer-secreted exosomes are critical mediators of cancer-host crosstalk. In the present study, we showed the delivery of miR-21-5p from colorectal cancer (CRC) cells to endothelial cells via exosomes increased the amount of miR-21-5p in recipient cells. MiR-21-5p suppressed Krev interaction trapped protein 1 (KRIT1) in recipient HUVECs and subsequently activated β-catenin signaling pathway and increased their downstream targets VEGFa and Ccnd1, which consequently promoted angiogenesis and vascular permeability in CRC. A strong inverse correlation between miR-21-5p and KRIT1 expression levels was observed in CRC-adjacent vessels. Furthermore, miR-21-5p expression in circulating exosomes was markedly higher in CRC patients than in healthy donors. Thus, our data suggest that exosomal miR-21-5p is involved in angiogenesis and vascular permeability in CRC and may be used as a potential new therapeutic target.

## Introduction

Colorectal cancer (CRC) is the third most common cancer worldwide^1^. Recurrence and metastasis are the leading cause of high mortality in CRC patients. Tumor growth and metastasis can, in great part, be due to angiogenesis and vascular permeability^2^. Anti-angiogenic therapy is one of the promising strategies for CRC. However, the primary or acquired resistance towards anti-angiogenic drugs results in little or no beneficial effect for some patients^3^. Therefore, there is a great and urgent need to identify a potential biomarker and new therapeutic target of anti-angiogenic therapy for CRC patients.

Exosomes, small membrane vesicles in the size range of ~40–160 nm (average ~100 nm) in diameter, are enriched in abundant proteins, lipids, and nucleic acids, like microRNAs (miRNAs)^4^. Exosomes released by various types of cells, including cancer cells, can be uptaken selectively by neighboring or distant cells^5,6^. In this way, molecules of exosomes can reprogram multiple cellular mechanisms in recipient cells to mediate intercellular communication^4,7^.

MiRNAs are a group of small non-coding RNAs with 18–25 nucleotides^8^. MiRNAs post-transcriptionally repress target genes by specifically binding to their 3′-untranslated regions (3′-UTRs), through which they regulate various biological processes^8^. Recent findings have noted that exosomal miRNAs derived from the serum of cancer patients are aberrantly expressed, and, to certain content, reflect miRNAs expression levels in the solid tumor^9^. Furthermore, exosomal miRNAs have been recognized as critical mediators of cancer-host crosstalk and are involved in the progression of cancer, such as modulation of immune response^10^, tumor microenvironment remodeling^11^, and pre-metastatic niche formation^6^. As contributing factors of tumor microenvironment, angiogenesis and vascular permeability are necessary for the continued survival and development of cancer^12,13^. Previous studies have shown that high expression of exosomal miR-92a-3p in serum was closely linked with metastasis and chemotherapy resistance in CRC patients^14^. Exosome-delivered miR-135b suppressed FOXO1 expression in recipient endothelial cells to promote angiogenesis in gastric cancer^15^. Exosomal miR-105, secreted by metastatic breast cancer cells, could be delivered to endothelial cells, where they destroyed vascular endothelial barriers to promote metastasis by targeting the tight junction protein zonula occludens 1 (ZO-1)^16^. Although much progress has been garnered in elucidating the molecular mechanisms that trigger cancer-induced angiogenesis and vascular permeability, many key mechanistic gaps remain to be explained.

MiR-21 acts as an oncogene in several human cancers, including ovarian cancer^17^, non-small cell lung cance^18^, and CRC^19,20^. It is identified to promote malignant biological behavior by directly targeting a range of tumor suppressor genes^18,20^. Although miR-21-5p and miR-21-3p are derived from a single precursor, previous studies about miR-21 have mainly focused on miR-21-5p probably because of its stability and functionality^17–21^. MiR-21 is highly expressed in CRC cells and promotes their proliferation, migration, and invasion^19,20^. Recent miRNA microarray analysis showed that miR-21 was significantly elevated in exosomes derived from the plasma of CRC patients and was associated with liver metastasis and TNM stage^22,23^. In addition, it has been shown that exosomes released by adipose-derived stem cells overexpressing miR-21 could potentially increase the tube formation capacity of recipient endothelial cells^24^. However, the specific roles and underlying mechanisms of exosomal miR-21-5p on vascular endothelial cells in CRC still remain unclear.

In the present study, we showed that miR-21-5p was overexpressed in CRC-adjacent vessels, in which miR-21-5p expression positively correlated with that in cancer epithelium. The transfer of exosomal miR-21-5p from CRC cells to endothelial cells elevated miR-21-5p expression in recipient cells. MiR-21-5p suppressed Krev interaction trapped protein 1 (KRIT1) and activated β-catenin signaling pathway to induce angiogenesis and vascular permeability. Moreover, an inverse correlation between miR-21-5p and KRIT1 expression was observed in CRC-adjacent vessels. MiR-21-5p was highly expressed in circulating exosomes of CRC patients than healthy donors. Thus, our data show that exosomal miR-21-5p may be used as a promising therapeutic target for angiogenesis and vascular permeability in CRC.

## Materials and methods

### Cell culture

Human umbilical vein endothelial cells (HUVEC), human embryonic kidney 293A cell line (HEK293A), human CRC cell lines Lovo, SW620, HT29, SW480, HCT116, and LS174T were obtained from Sun Yat-sen University (Guangzhou, China). The above cell lines were cultured in DMEM (GIBCO, Gaithersburg, MD, USA) (HUVEC) or RPMI-1640 (GIBCO, Gaithersburg, MD, USA) (HEK293A and human CRC cell lines) medium with 10% fetal bovine serum (FBS) (HyClone, Logan, USA) in 5% CO_2_ at 37 °C. Cell lines were authenticated by STR profiling, and tested negative for mycoplasma before experiments.

### Clinical samples

All primary or metastatic CRC tissue samples and matched normal tissue samples were collected from patients who received CRC resection with no previous chemoradiotherapy and radiotherapy in Dongguan people’s Hospital. Serum samples from healthy donors and CRC patients were obtained from Dongguan people’s Hospital and stored at –80 °C until needed. Controls were selected from age, sex, and race-matched healthy donors. Serum specimens were collected from CRC patients that received a radical operation on the day of operation and 7 days after the operation. All clinical samples were obtained the informed consent of patients or volunteers and approved by the ethics committee of Dongguan People’s Hospital (ethics number: PJ201711-016; PJ201711-017).

### Exosomes isolation, characterization, and treatment

CRC-derived conditioned medium was obtained from cells cultured in RPMI-1640 medium added with 10% exosome-depleted FBS (ABW, Gaithersburg, MD, USA) for 48 h, and remove cells and debris by centrifugation. Exosomes were purified from serum of CRC patients or conditioned medium by ultra-centrifugation and resuspended in PBS^6^. The particle sizes of exosomes were measured by ZetaView (Particle Metrix, Germany). Exosomes were observed on transmission electron microscope (Hitachi H-7500, Japan). For quantification of exosomes, the amount of exosomes was measured via BCA Protein Assay kit (KeyGEN BioTECH, Nanjing, China). For cell treatment, 2 × 10^5^ receptor cells were added with 2 µg exosomes for 48 h.

### Vectors construction and retroviral infection

pPACKH1 lentivector Packaging Kit (System Biosciences, USA) was applied to generate lentiviral constructs expressing miR-21-5p or repressing miR-21-5p. Lentiviral constructs were used to infect CRC cells to establish cells consistently and steadily expressing miR-21-5p or repressing miR-21-5p. In the rescue experiments, HUVECs that co-cultured with SW480/miR-21-5p exosomes were transfected with the KRIT1 expressing plasmids lacking 3′UTR (GeneCopoeia, USA), and HUVECs that incubated with SW620/zip-miR-21-5p exosomes were transfected with siRNAs towards KRIT1 (System Biosciences, USA).

### Quantitative real-time PCR (qRT-PCR)

Total RNA was extracted from cultured cells or exosomes using Trizol reagent (Invitrogen, Carlsbad, CA, USA) according to the manufacturer’s protocol. MiRNA or mRNA was synthesized into cDNA with miRNA-specific RT primers (RiboBio, Guangzhou, China) or PrimeScript RT Master Mix (Takara, Dalian, China). Real-time polymerase chain reaction (RT-qPCR) was performed on an ABI Prism 7900 System (Applied Biosystems, CA, USA) using SYBR Green Premix Ex Taq (Takara, Dalian, China). Relative gene expression levels were calculated using the 2-comparative Ct (2^−ΔΔCτ^) method and normalized to the amount of GAPDH for mRNA or of RNU6B for miRNA. The sequences of indicated primers were listed in Supplementary Table 1.

### Cell proliferation assay

The pre-processed HUVECs were cultured in 96-well plates, then analyzed by CCK8 (Dojindo, Japan) according to the manufacturer’s instructions, and the absorbance at the wavelength of 450 nm was simultaneously read every day using an enzyme labeling instrument (Sunrise, Tecan, Austria) for 7 days.

### Cell migration assay

Cell migration assay was performed using transwell chambers (catalog no. 3422; Corning-Costar, NY, USA). DMEM with 15% FBS was placed in the bottom of the chambers. 1 × 10^5^ HUVECs were placed in the upper chamber, followed by the addition of DMEM with 5% FBS and 1 μg exosomes. 10 h later, the chamber was fixed with formalin, and the cells in the upper chamber were erased. The cells passing through the membrane were counted under a microscope (Olympus, Tokyo, Japan) after Hematoxylin-Eosin (HE) staining.

### Tube formation assay

For tube formation assay, 24-well plates were added with matrigel matrix (Corning, NY, USA) and polymerized for 30 min at 37 °C. The pre-processed HUVECs were loaded on the top of the matrigel (15,000 cells per well), then cultured at suitable growth conditions (37 °C; 5% CO_2_) for 12 h. The formed tubes were counted under a microscope (Olympus, Tokyo, Japan).

### In vitro permeability assay

For in vitro permeability assay, the pre-treated HUVECs were plated and allowed to reach confluence on the top of the transwell membrane (diameter: 0.4 μm; Corning-Costar, NY, USA). Subsequently, rhodamine-dextran (average MW ~70,000; Sigma, USA) was added in the upper chamber at 20 mg/ml for 30 min. 40 μl medium of the lower chamber was absorbed and measured under 544 nm excitation and 590 nm emission.

### Trans-endothelial invasion assay

For trans-endothelial invasion assay, the pre-treated HUVECs were seeded and allowed to grow a monolayer on the top of the transwell membrane (diameter: 8.0 μm; Corning-Costar, NY, USA). GFP-labeled CRC cells (with pBABE-GFP retrovirus) were plated in the upper chamber (50,000 cells per well). After 10 h, cells in the upper chamber were scraped off, and the GFP+ cells passing through the membrane were counted under an inverted fluorescent microscope (Olympus, Center Valley, PA).

### Luciferase activity assay

For luciferase reporter assays, the 3′UTR segments of human or mouse KRIT1 containing miR-21-5p binding sites were amplified by PCR and inserted into the psiCHECK2 vector. Primers of human KRIT1 3’UTR were as described previously^25^, and primers of mouse KRIT1 3′UTR were designed using Primer Premier 5.0. The KRIT1 3′UTR plasmids were co-transfected with mimics or inhibitor of miR-21-5p into the cells using Lipofectamine 3000 (Invitrogen, Carlsbad, CA, USA). After 48 h, luciferase activity was measured by the Dual-Luciferase Reporter Assay Kit (Promega, Madison, WI, USA).

### In situ hybridization (ISH)

Tissues were sliced into 3 μm thick after conventional fixation and embedding. Digoxigenin-labeled miR-21-5p probe (Bosterbio, USA, 1:100 dilution) were used to carry out ISH of CRC tissues following established protocols^6,16^. The intensity of staining was scored as -: 0; +: 1; ++: 2; +++: 3 and percentage of the cells of interest staining positive for each antigen was scored as 0%: 0; 1 ~ 29%: 1; 30′~ 69%: 2; and ≥ 70%: 3. The intensity score was multiplied by the percentage score to obtain a final score.

### Immunohistochemistry (IHC)

Tissues were sliced into 3 μm thick after conventional fixation and embedding. Immunohistochemistry of CRC tissues were performed following established protocols^6,16^. The intensity of staining was scored as -: 0; +: 1; ++: 2; +++: 3 and percentage of the cells of interest staining positive for each antigen was scored as 0%: 0; 1 ~ 29%: 1; 30 ~ 69%: 2; and ≥ 70%: 3. The intensity score was multiplied by the percentage score to obtain a final score. The following primary antibodies were used: anti-CD31(Proteintech, 66065-1-Ig, 1:1000 dilution), anti-human KRIT1 (Abcam, ab111504, 1:40 dilution), and anti-mouse KRIT1 (Abcam, ab 187988, 1:100 dilution).

### Immunofluorescence

Immunofluorescence of cells was carried out according to established protocols^6,8^. Immunofluorescence images were captured by an inverted fluorescence microscope (Olympus; Center Valley, PA) and were outputted by Image J software. The following primary antibodies were used: anti-β-catenin (Cell Signaling Technology, 2677S, 1:150 dilution).

### Western blotting

Western blotting was performed according to standard protocols^6,8^. The following primary antibodies were used: anti-KRIT1 (Abcam, ab126191, 1:800 dilution), anti-TSG101 (Abcam, ab83, 1:500 dilution), anti-GM130 (Abcam, ab187514, 1:400 dilution) and anti-β-actin (Abcam, ab8227, 1:1000 dilution).

### Chick chorioallantoic membrane (CAM) assay

Fertilized chicken eggs were incubated at 37 °C for 9 days under 70% humidity. Then the embryo growth was confirmed and vascularized regions of the CAM were located. A hole was drilled. 3 cm × 4 cm regions of the CAM were exposed and inoculated with exosomes prior to incubation for the other 9 days. Lastly, the exposed regions of CAMs were dissected, washed, and photographed.

### Animal models

The male athymic BALB/c-nu/nu mice (4-to-6-weeks old) were purchased from the Central Laboratory of Animal Science of Southern Medical University (Guangzhou, China), and maintained in a specific Pathogen Free environment. All procedures performed on the animals were approved by the animal ethics committee of Guangdong Medical University. For the orthotopic xenografts model, 2 × 10^6^ CRC cells were injected into the mesentery of the caecum in anesthetized nude mice. After 2 months, mice were sacrificed for tissues or blood collection. For in vivo vascular permeability assay, exosomes were injected into the tail vein of nude mice at 2 μg per injection twice a week. After five injections, 100 mg/kg rhodamine-dextran (Sigma, USA) was intravenously injected into the tail vein of nude mice. Intracardiac perfusion was carried out to eliminate the excessive dye 3 h later, and all organs were excised for examination. For tail vein metastasis assay, exosomes were injected into the tail vein of nude mice at 2 μg per injection twice a week. After five injections, 2 × 10^6^ SW480 were injected into the mice through the tail vein. Mice were sacrificed 2 months later, and lung tissues were removed for biopsy. Images were captured using Olympus DP72 upright microscope and were outputted by DP2-BSW software.

### Statistical analysis

All assays were performed at least in triplicate and each experiment was repeated three times. All data were presented as the mean ± SEM, which were calculated from three independent experiments. Statistical significance among/between groups was examined with one-way analysis of variance (ANOVA) or independent-samples *t*-test. Spearman’s correlation coefficient was applied to estimate the degree of the linear relationship of gene expression levels. All statistical analyses were conducted with SPSS 19.0 statistical software. *P* < 0.05 was considered to be statistically significant.

## Results

### Expression and localization of miR-21-5p in CRC

**Fig. 1:**
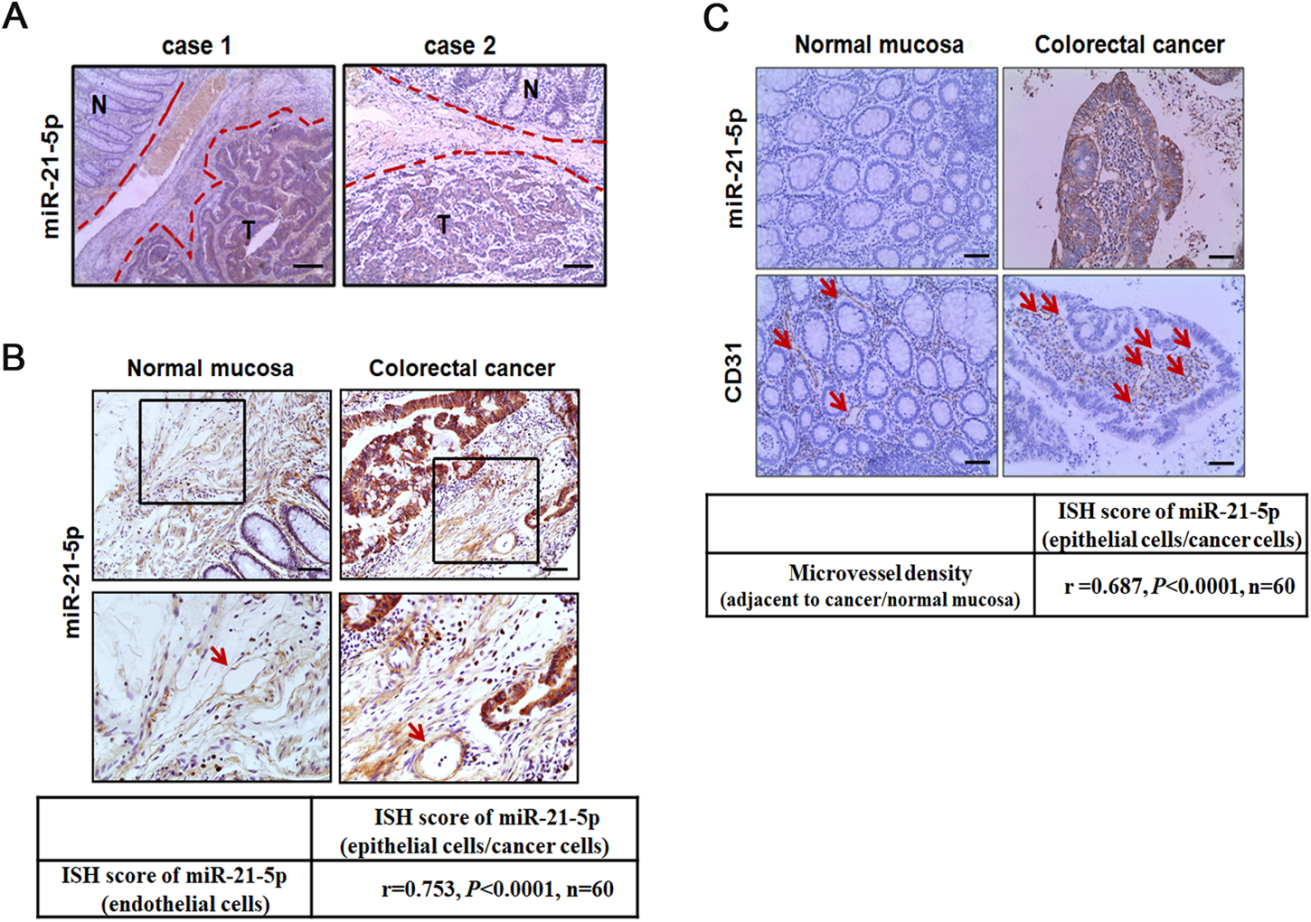
Expression and localization of miR-21-5p in CRC. **A** Expression of miR-21-5p in primary CRC tissues (T) and matched adjacent normal mucosa (N) by ISH (*n* = 60). Scale bars represent 100 µm. **B** Expression of miR-21-5p in vessels (indicated by arrows) adjacent to primary CRC tissues and matched adjacent normal mucosa by ISH. Correlation analysis of miR-21-5p expression in colorectal epithelial cells/cancer cells and their adjacent endothelial cells. MiR-21-5p levels were scored as described in “Methods”. Spearman’s correlation coefficient (*r*) and *P*-value are shown (*n* = 60). Scale bars in top panels represent 50 µm. **C** Correlation analysis of miR-21-5p expression in colorectal epithelial cells/cancer cells and their adjacent microvessel density (labeled by CD31 and indicated by arrows). Microvessel density was scored as described in Methods. Spearman’s correlation coefficient (*r*) and *P*-value are shown (*n* = 60). Scale bars represent 50 µm.

MiR-21-5p has been proved to be an oncogene and facilitates cancer epithelial cells proliferation, migration, and invasion, leading to a poor prognosis in CRC^19,20,26^. To better understand the function of miR-21-5p in CRC, the expression and localization of miR-21-5p were evaluated by in situ hybridization (ISH) in 60 paired CRC tissues. The result revealed that miR-21-5p was expressed in both CRC epithelium and stromal cells, including fibroblasts^27^ and endothelial cells^28^ as previously reported. MiR-21-5p was overexpressed in CRC than matched adjacent normal mucosa (Fig. 1A). Notably, miR-21-5p expression was higher in cancer-adjacent vessels than normal mucosa-adjacent vessels (Fig. 1B). Across all clinical specimens, miR-21-5p expression levels in cancer-adjacent vessels positively correlated with that in cancer epithelium (*r* = 0.753, *P* < 0.0001, Fig. 1B). Even more, we detected a strong positive correlation between miR-21-5p expression levels in cancer epithelium and microvessel density (MVD) in CRC (*r* = 0.687, *P* < 0.0001, Fig. 1C). The above results suggest a possible link among CRC, miR-21-5p, and CRC-adjacent vessels.

### CRC-secreted miR-21-5p is transferred to endothelial cells via exosomes

**Fig. 2:**
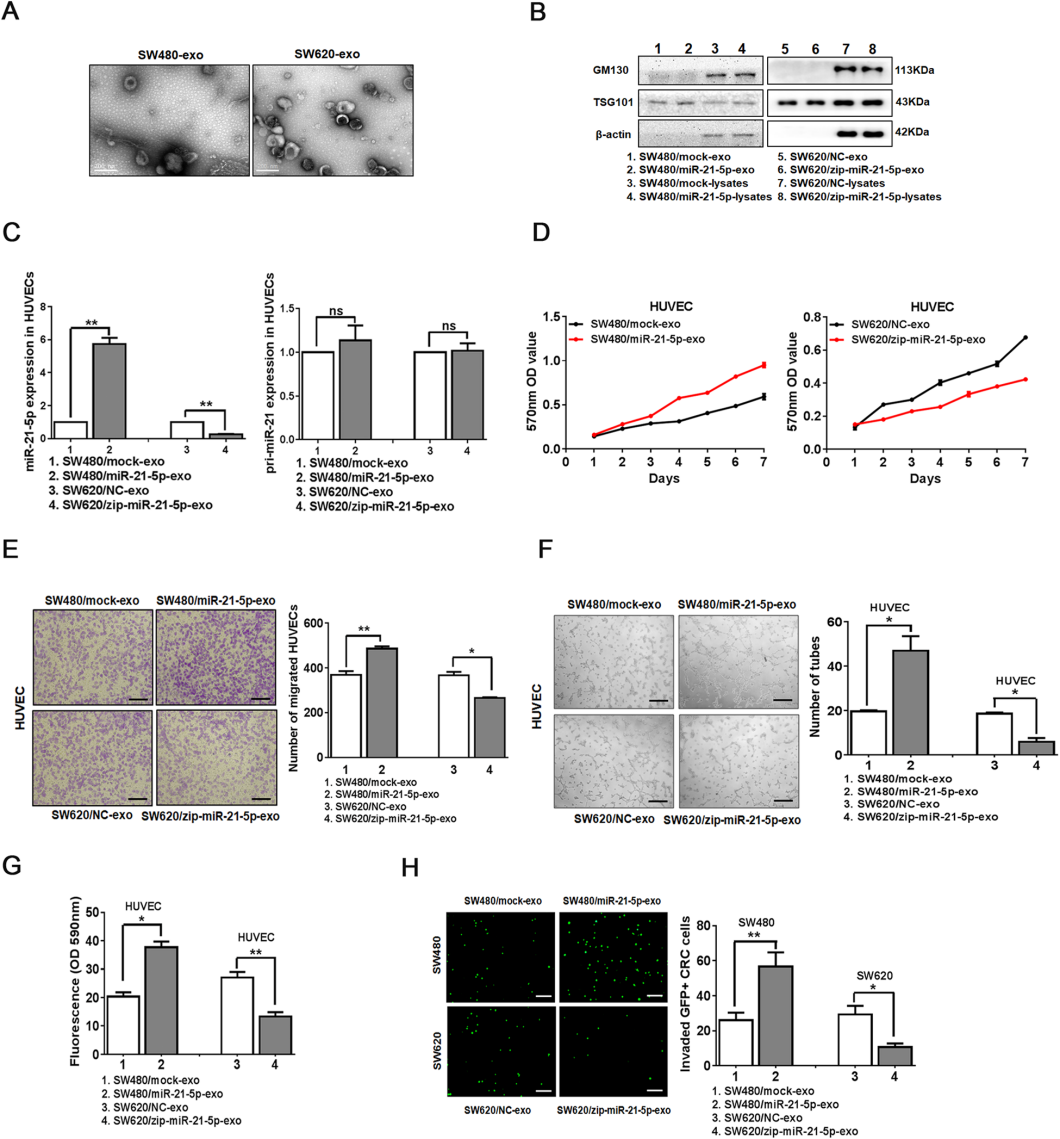
CRC-secreted exosomal miR-21-5p is transferred to endothelial cells and induces angiogenesis and vascular permeability in vitro. **A** Transmission electron microscopy of exosomes derived from SW480 and SW620. Scale bars represent 200 nm. **B** Expression of GM130 and TSG101 in cell lysates or exosomes extracted from SW480/mock, SW480/miR-21-5p, SW620/NC, or SW620/zip-miR-21-5p by Western blot. Expression levels were normalized to β-actin. **C** Expression of miR-21-5p and pri-miR-21 in HUVECs incubated with exosomes derived from SW480/mock, SW480/miR-21-5p, SW620/NC, or SW620/zip-miR-21-5p by qRT-PCR. Mean ± SEM are provided (*n* = 3). **D** Effect of SW480/mock exosomes, SW480/miR-21-5p exosomes, SW620/NC exosomes, and SW620/zip-miR-21-5p exosomes on the proliferation of HUVECs. Mean ± SEM are provided (*n* = 3). **E** Effect of SW480/mock exosomes, SW480/miR-21-5p exosomes, SW620/NC exosomes, and SW620/zip-miR-21-5p exosomes on the migration of HUVECs by Boyden chamber. Scale bars represent 50 µm. Mean ± SEM are provided (*n* = 3). **F** Effect of SW480/mock exosomes, SW480/miR-21-5p exosomes, SW620/NC exosomes, and SW620/zip-miR-21-5p exosomes on tube formation ability of HUVECs. Scale bars represent 50 µm. Mean ± SEM are provided (*n* = 3). **G** Effect of SW480/mock exosomes, SW480/miR-21-5p exosomes, SW620/NC exosomes, and SW620/zip-miR-21-5p exosomes on the permeability of the HUVEC monolayers to rhodamine-dextran by in vitro permeability assay. Mean ± SEM are provided (*n* = 3). **H** The number of GFP + CRC cells invaded through the HUVEC monolayers pre-treated with SW480/mock exosomes, SW480/miR-21-5p exosomes, SW620/NC exosomes, and SW620/zip-miR-21-5p exosomes. Scale bars represent 50 µm. Mean ± SEM are provided (*n* = 3). *P < 0.05, **P < 0.01, ***P < 0.001. NS represents no significant difference.

Recently, miRNAs can be delivered between cells via exosomes to mediate intercellular communication^7^. MiRNA microarray analysis detected a significant increase of miR-21 expression in circulating exosomes derived from CRC patients^29^. Based on previous studies and the clinical observations above, we speculated whether exosomal miR-21-5p secreted by CRC cells could be delivered to endothelial cells and regulate their biological functions. Therefore, we firstly detected endogenous miR-21-5p expression in 6 CRC cell lines by qRT-PCR (Supplementary Fig. 1A). Then we stably overexpressed miR-21-5p in SW480, and knockdown miR-21-5p in SW620 and SW480 (Supplementary Fig. 1B), leading to the corresponding up-regulation or downregulation of miR-21-5p in exosomes, respectively (Supplementary Fig. 1C). The exosomes exhibited typical cup-shaped particles in the size range of 40 to 160 nm in diameter, as confirmed by the particle size distribution and transmission electron microscopy imaging (Supplementary Fig. 1D, Fig. 2A). The expression of the exosome marker TSG101, but not the cellular marker GM130, was detected in exosome lysates, confirming that there was no cellular contamination in isolated exosomes (Fig. 2B). To explore whether miR-21-5p secreted by CRC cells could be transferred to endothelial cells via exosomes, we analyzed the expression of miR-21-5p and pri-miR-21 in HUVECs incubated with different CRC-derived exosomes. Interestingly, compared with corresponding control group, miR-21-5p was highly expressed in HUVECs treated with SW480/miR-21-5p exosomes, while miR-21-5p was lowly expressed in HUVECs treated with SW620/zip-miR-21-5p exosomes or SW480/zip-miR-21-5p exosomes. However, there was no significant difference of pri-miR-21 expression between the two groups (Fig. 2C, Supplementary Fig. 1E). Since miR-21-5p is generated from pri-miR-21 transcripts containing stem-loop structure^30^, the above results indicated that it was the delivery of mature miR-21-5p, but not other molecules of the exosome that increased miR-21-5p expression in the recipient cells. Thus, our data uncover that CRC-secreted miR-21-5p is delivered to endothelial cells via exosomes.

### CRC-secreted exosomal miR-21-5p induces angiogenesis and vascular permeability

To investigate the role of exosomal miR-21-5p in endothelial cells, we incubated HUVECs with miR-21-5p-rich or miR-21-5p-deficient exosomes. Compared with corresponding negative controls, treatment with SW480/miR-21-5p exosomes strikingly induced the proliferation and migration of HUVECs, while treatment with SW620/zip-miR-21-5p exosomes or SW480/zip-miR-21-5p exosomes markedly suppressed the proliferation and migration of HUVECs (Fig. 2D, E, Supplementary Fig. 2A, B). Moreover, treatment with SW480/miR-21-5p exosomes obviously enhanced the ability of tube formation and vascular leakiness of HUVECs, but treatment with SW620/zip-miR-21-5p exosomes led to inverse effects (Fig. 2F, G). Trans-endothelial invasion assay was performed to directly simulate the vascular infiltration by tumor cells. An increase in the number of GFP-labeled SW480 that invaded through the HUVECs treated with SW480/miR-21-5p exosomes was observed compared to the number of those that invaded through the HUVECs treated with control exosomes. Conversely, SW620/zip-miR-21-5p exosomes or SW480/zip-miR-21-5p exosomes showed the opposite effects (Fig. 2H, Supplementary Fig. 2C).

**Fig. 3:**
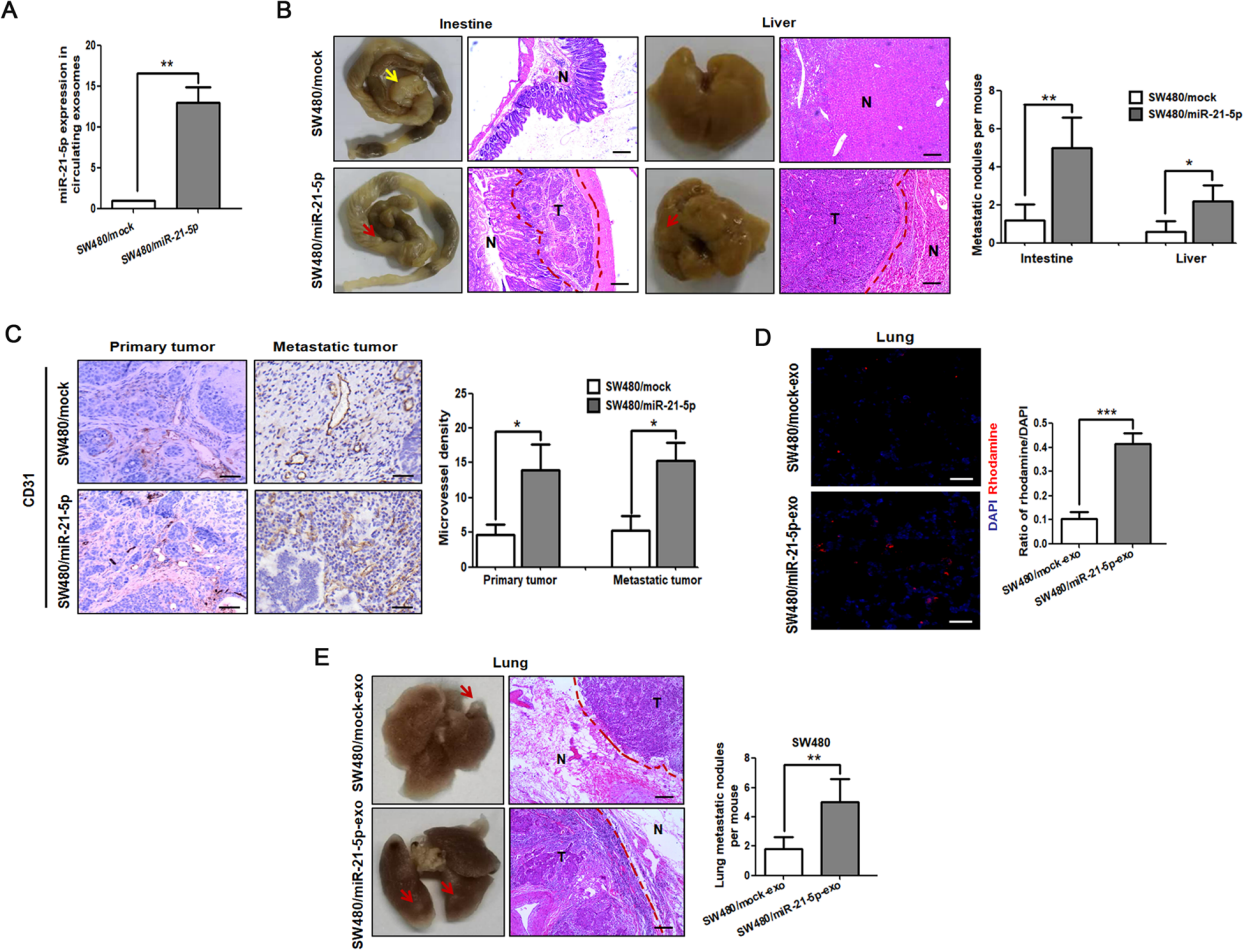
CRC-secreted exosomal miR-21-5p induces angiogenesis and vascular permeability in vivo. **A** Expression of miR-21-5p in circulating exosomes extracted from the serum of mice bearing SW480/mock or SW480/miR-21-5p tumors. Mean ± SEM are provided (*n* = 5 mice per group). **B** Intestinal and hepatic metastatic nodules after SW480/mock or SW480/miR-21-5p cells were injected in the cecal mesentery of nude mice for two months. Yellow arrow points at primary tumor, while red arrows point at metastatic nodules. Metastatic tumor tissues (T) and normal tissues (N) were observed. Scale bars represent 100 µm. The number of intestinal and hepatic metastatic nodules per mouse was counted under the microscope. Mean ± SEM are provided (*n* = 5 mice per group). **C** Introtumoral microvessel density in primary and metastatic tumors after SW480/mock or SW480/miR-21-5p cells were injected in the cecal mesentery of nude mice for two months. The number of microvessels was counted under the microscope (labeled by CD31). Scale bars represent 50 µm. Mean ± SEM are provided (*n* = 5 mice per group). **D** Effect of SW480/mock exosomes and SW480/miR-21-5p exosomes on vascular permeability of lung in nude mice to rhodamine-dextran by in vivo vascular permeability assay. Rhodamine-dextran was injected into the tail vein of nude mice pre-treated with SW480/mock exosomes or SW480/miR-21-5p exosomes. Levels of rhodamine-dextran fluorescence in tissues were quantified using Image J software and normalized to the levels of DAPI. Scale bars represent 50 µm. Mean ± SEM are provided (*n* = 5 mice per group). **E** SW480 were injected into the tail vein of nude mice pre-treated with SW480/mock exosomes or SW480/miR-21-5p exosomes. Red arrows point at lung metastatic nodules. Metastatic tumor tissues (T) and normal tissues (N) were observed. Scale bars represent 100 µm. The number of lung metastatic nodules per mouse was counted under the microscope. Mean ± SEM are provided (*n* = 5 mice per group). *P < 0.05, **P < 0.01, ***P < 0.001.

To further evaluate whether exosomal miR-21-5p could regulate angiogenesis and vascular permeability in vivo, we firstly established orthotopic xenografts model by injecting SW480 cells with or without miR-21-5p overexpression into the mesentery at the tail end of the cecum. Compared with the control group, mice bearing miR-21-5p overexpressing tumors secreted a higher level of miR-21-5p in circulating exosomes and strikingly enhanced their capacity to seed spontaneous intestinal and liver metastases (Fig. 3A, B). Overexpression of miR-21-5p enhanced primary tumor growth, but the size of metastatic nodules showed no difference between the two groups (Supplementary Fig. 2D). Noticeably, compared with SW480/mock tumors, an increase in the number of vessels was observed in SW480/miR-21-5p tumors, including primary and metastatic tumors (Fig. 3C). Chick chorioallantoic membrane assay showed that SW480/miR-21-5p exosomes could effectively increased the number of new vessels, while SW620/zip-miR-21-5p exosomes had a reverse effect (Supplementary Fig. 2E). For in vivo vascular permeability assay, rhodamine-dextran was injected into the circulation of nude mice pre-treated with SW480/miR-21-5p exosomes or SW480/mock exosomes. Our result showed that the rhodamine-dextran was leaked into the lung tissues of nude mice pre-treated with SW480/miR-21-5p exosomes, whereas little or no dye was observed in the lung tissues of nude mice pre-treated with control exosomes (Fig. 3D). To evaluate whether exosomal miR-21-5p could affect the vascular barrier-traversing step in metastasis, we inoculated SW480 into the circulation of nude mice pre-injected with SW480/miR-21-5p exosomes or control exosomes. Mice treated with SW480/miR-21-5p exosomes showed a significant increase in the number of lung metastasis as compared to the control group (Fig. 3E). Taken together, the above results suggest that exosomal miR-21-5p derived from CRC cells induces angiogenesis and vascular permeability in vitro and in vivo.

### Exosomal miR-21-5p induces angiogenesis and vascular permeability by regulating KRIT1

**Fig. 4:**
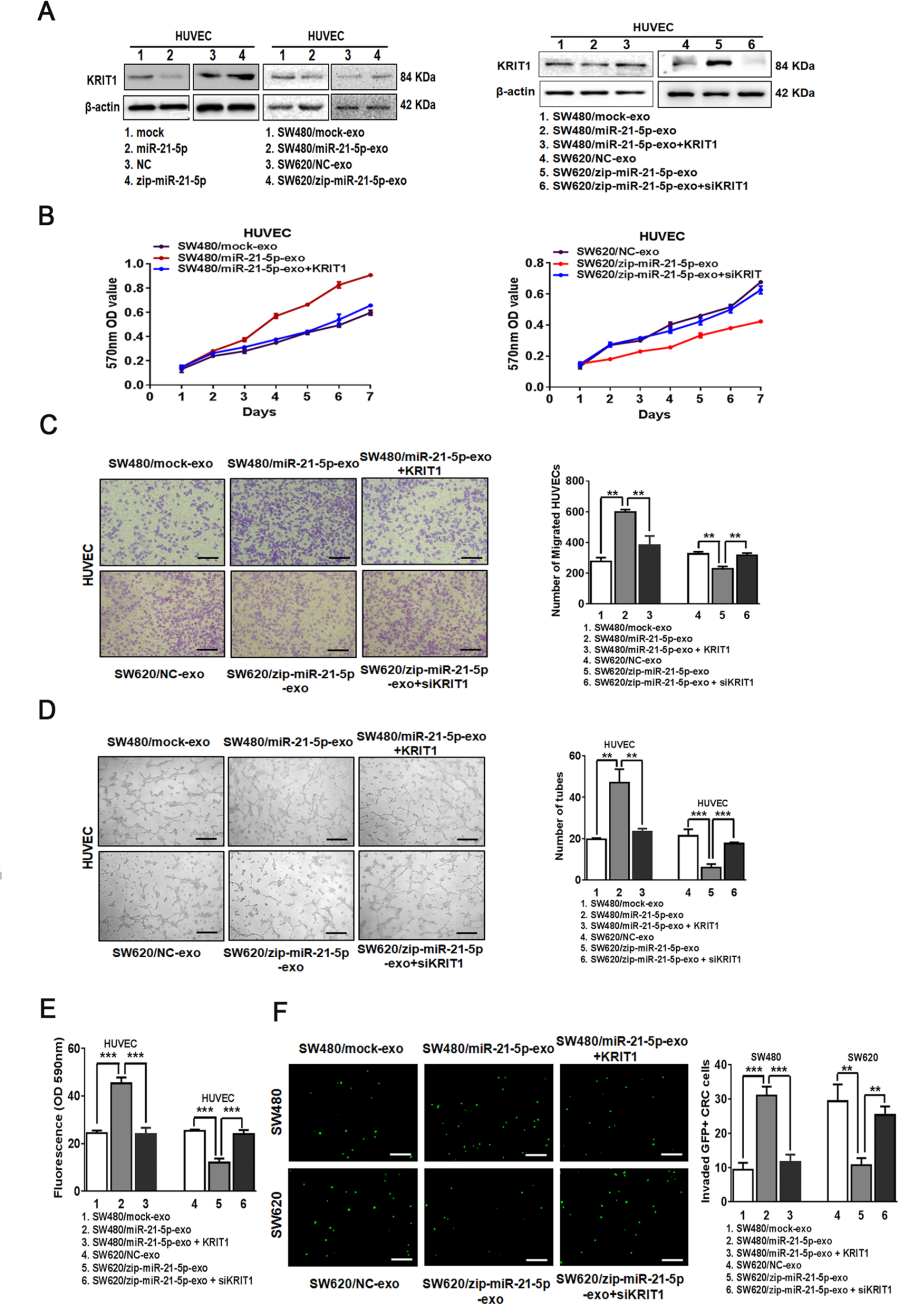
Exosomal miR-21-5p induces angiogenesis and vascular permeability by regulating KRIT1. **A** Expression of KRIT1 in HUVECs treated with miR-21-5p mimics, miR-21-5p inhibitor, SW480/mock exosomes, SW480/miR-21-5p exosomes, SW620/NC exosomes, SW620/zip-miR-21-5p exosomes, SW480/mock exosomes, SW480/miR-21-5p exosomes, SW480/miR-21-5p exosomes+KRIT1, SW620/NC exosomes, SW620/zip-miR-21-5p exosomes or SW620/zip-miR-21-5p exosomes+siKRIT1 by Western blot. Expression levels were normalized to β-actin. **B** Effect of SW480/mock exosomes, SW480/miR-21-5p exosomes, SW480/miR-21-5p exosomes+KRIT1, SW620/NC exosomes, SW620/zip-miR-21-5p exosomes and SW620/zip-miR-21-5p exosomes+siKRIT1 on the proliferation of HUVECs. Mean ± SEM are provided (*n* = 3). **C** Effect of SW480/mock exosomes, SW480/miR-21-5p exosomes, SW480/miR-21-5p exosomes+KRIT1, SW620/NC exosomes, SW620/zip-miR-21-5p exosomes and SW620/zip-miR-21-5p exosomes+siKRIT1 on the migration of HUVECs by Boyden chamber. Scale bars represent 50 µm. Mean ± SEM are provided (*n* = 3). **D** Effect of SW480/mock exosomes, SW480/miR-21-5p exosomes, SW480/miR-21-5p exosomes+KRIT1, SW620/NC exosomes, SW620/zip-miR-21-5p exosomes and SW620/zip-miR-21-5p exosomes+siKRIT1 on tube formation ability of HUVECs. Scale bars represent 50 µm. Mean ± SEM are provided (*n* = 3). **E** Effect of SW480/mock exosomes, SW480/miR-21-5p exosomes, SW480/miR-21-5p exosomes+KRIT1, SW620/NC exosomes, SW620/zip-miR-21-5p exosomes and SW620/zip-miR-21-5p exosomes+siKRIT1 on the permeability of the HUVEC monolayers to rhodamine-dextran by in vitro permeability assay. Mean ± SEM are provided (*n* = 3). **F** The number of GFP + CRC cells invaded through the HUVEC monolayers pre-treated with SW480/mock exosomes, SW480/miR-21-5p exosomes, SW480/miR-21-5p exosomes+KRIT1, SW620/NC exosomes, SW620/zip-miR-21-5p exosomes or SW620/zip-miR-21-5p exosomes+siKRIT1. Scale bars represent 50 µm. Mean ± SEM are provided (*n* = 3). *P < 0.05, **P < 0.01, ***P < 0.001.

To explore the effectors of miR-21-5p in endothelial cells, we predicted the downstream targets of miR-21-5p by four mRNA target-predicting algorithms (miRanda, miRDB, miRWalk, and Targetscan) and focused on those related to vascular function. Among all the potential targets, KRIT1 has been reported to be a direct target of miR-21 and a crucial regulator of vessel formation and vascular integrity^25^. Consistent with a previous study^25^, reporter assays revealed that miR-21-5p repressed the luciferase activity of human KRIT1 3′UTR in HUVECs and HEK293A (Supplementary Fig. 3A). In agreement with these observations, miR-21-5p ectopic expression or treatment with SW480/miR-21-5p exosomes in HUVECs decreased the expression of KRIT1, while knockdown of miR-21-5p or treatment with SW620/zip-miR-21-5p exosomes in HUVECs increased KRIT1 expression (Fig. 4A).

To identify whether restoration of KRIT1 could reverse exosomal miR-21-5p-induced biological changes of endothelial cells, we incubated KRIT1 over-expressing HUVECs with SW480/miR-21-5p exosomes or incubated KRIT1 depleting HUVECs with SW620/zip-miR-21-5p exosomes, then confirmed its expression (Supplementary Fig. 3B, Fig. 4A). Treatment with SW480/miR-21-5p exosomes promoted the proliferation and migration of HUVECs in vitro, while restoration of KRIT1 in HUVECs abrogated these effects (Fig. 4B, C). KRIT1 rescued exosomal miR-21-5p-dependent promoting effects on tube-formation and the vascular leakiness (Fig. 4D, E). Furthermore, treatment with SW480/miR-21-5p exosomes in HUVECs increased the number of trans-endothelial invasion of CRC cells, whereas overexpression of KRIT1 in recipient HUVECs could reverse the effect (Fig. 4F). Similarly, the same results were achieved in KRIT1 depleting HUVECs incubated with SW620/zip-miR-21-5p exosomes (Fig. 4B–F). In addition, KRIT1 gene is highly homologous in two mammalian species, human and mouse. The sequences of the conserved binding sites of miR-21-5p in human and mouse KRIT1 3′UTR only differ by one base (Supplementary Fig. 3C, TargetScan 7.0). The luciferase activities of mouse KRIT1 3′UTR were also suppressed by hsa-miR-21-5p in HEK293A (Supplementary Fig. 3D). Even more, KRIT1 expression was obviously decreased in vessels adjacent to primary CRC than those adjacent to normal mucosa in SW480/miR-21-5p tumors of orthotopic xenografts model (Supplementary Fig. 3E), which were consistent with the role of miR-21-5p in downregulating KRIT1. Thus, these results make it clear that KRIT1 is necessary for miR-21-5p-mediated angiogenesis and vascular permeability.

**Fig. 5:**
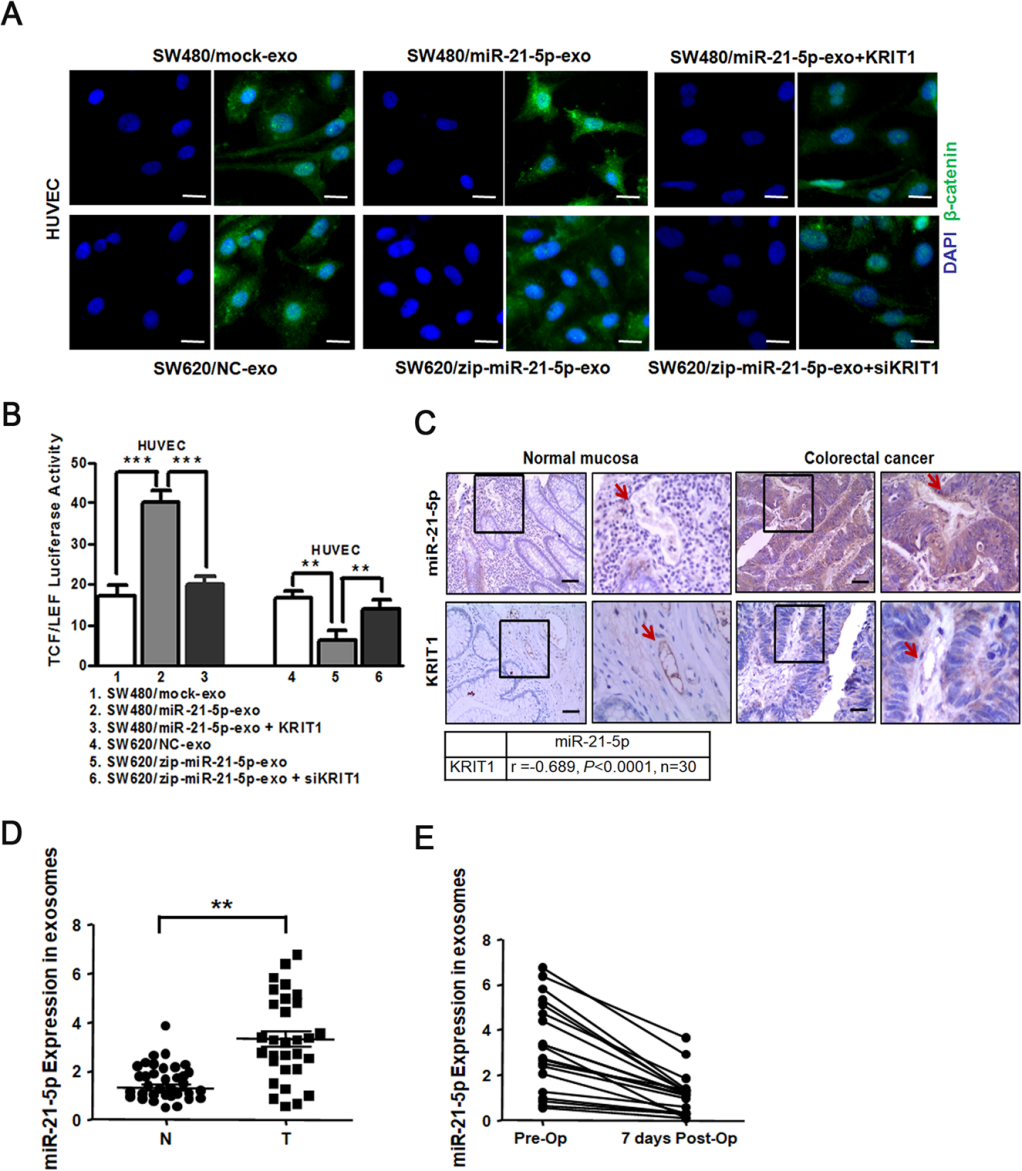
Correlations of miR-21-5p with KRIT1 expression in CRC-adjacent vessels. **A** Immunofluorescence images of nuclear translocation of β-catenin in HUVECs treated with SW480/mock exosomes, SW480/miR-21-5p exosomes, SW480/miR-21-5p exosomes+KRIT1, SW620/NC exosomes, SW620/zip-miR-21-5p exosomes or SW620/zip-miR-21-5p exosomes+siKRIT1. Scale bars represent 5 µm. **B** Luciferase activities of TCF/LEF transcription in HUVECs treated with SW480/mock exosomes, SW480/miR-21-5p exosomes, SW480/miR-21-5p exosomes+KRIT1, SW620/NC exosomes, SW620/zip-miR-21-5p exosomes or SW620/zip-miR-21-5p exosomes+siKRIT1. Mean ± SEM are provided (*n* = 3). **C** Correlation analysis between miR-21-5p and KRIT1 expression in vessels (indicated by arrows) adjacent to primary CRC tissues or matched normal mucosa. MiR-21-5p levels were scored by ISH, while KRIT1 levels were scored by IHC. Spearman’s correlation coefficient (*r*) and *P*-value are shown (*n* = 30). Scale bars represent 50 µm. **D** Expression of miR-21-5p in exosomes derived from the serum of CRC patients (T) and healthy donors (N) (N = T = 30). **E** Expression of miR-21-5p in exosomes derived from the serum of CRC patients that received a radical operation on the day of operation (Pre-Op) and 7 days after the operation (7 days Post-Op) (*n* = 20). *P < 0.05, **P < 0.01, ***P < 0.001.

Several studies have pointed out that KRIT1 mediates the stabilization of β-catenin-containing endothelial cell-cell junctions^31^. β-catenin plays a structural role in cell adhesion by binding to cadherins at the intracellular surface of the plasma membrane, and signaling through β-catenin is regulated by modulating its degradation, nuclear translocation, and TCF/LEF transcription^32^. To investigate whether the β-catenin signaling pathway was necessary for exosomal miR-21-5p-induced angiogenesis and vascular permeability, we detected the activity of β-catenin signaling pathway among groups. We found that treatment with SW480/miR-21-5p exosomes in HUVECs led to nuclear translocation of β-catenin (Fig. 5A), TCF/LEF transcriptional activation (Fig. 5B), and up-regulation of downstream angiogenesis-related target genes VEGFa and Ccnd1 (Supplementary Fig. 3F), while these effects could be rescued by reintroduction of KRIT1 in recipient HUVECs (Fig. 5A, B, Supplementary Fig. 3F). The same results were confirmed in KRIT1 depleting HUVECs treated with SW620/zip-miR-21-5p exosomes (Fig. 5A, B, Supplementary Fig. 3F). Collectively, the results reveal that exosomal miR-21-5p induces angiogenesis and vascular permeability via the β-catenin signaling pathway.

### Correlations of miR-21-5p with KRIT1 expression in CRC-adjacent vessels

To determine whether miR-21-5p could functionally affect KRIT1 expression in clinical specimens, we detected the expression levels of miR-21-5p and KRIT1 in a matched collection of 30 human CRC tissues. Compared with normal mucosa-adjacent vessels, miR-21-5p was markedly up-regulated, while KRIT1 was obviously downregulated in CRC-adjacent vessels. In addition, a negative correlation between miR-21-5p and KRIT1 expression levels was observed in CRC-adjacent vessels (*r* = −0.689, *P* < 0.0001, Fig. 5C). Then we also examined the expression levels of KRIT1 in 30 cases of paired liver metastatic CRC tissues. Similarly, KRIT1 was significantly downregulated in vessels adjacent to liver metastases from CRC than in those adjacent to the matched liver (Supplementary Fig. 3G). These observations are consistent with the role of miR-21-5p in downregulating KRIT1.

To ascertain whether miR-21-5p expression in circulating exosomes correlated with CRC, we detected miR-21-5p expression in circulating exosomes derived from serum of CRC patients or healthy donors. MiR-21-5p expression in circulating exosomes was higher in CRC patients than in healthy donors (Fig. 5D). We also extracted circulating exosomes from CRC patients before and after surgical resection of tumors, respectively. MiR-21-5p expression in circulating exosomes significantly decreased when CRC patients underwent surgical resection 7 days later (Fig. 5E). These data verify that miR-21-5p in circulating exosomes can be a potential non-invasive biomarker to evaluate angiogenesis and vascular permeability in CRC.

## Discussion

Growing evidence indicates that miR-21 plays oncogenic roles in various human cancers, including CRC^19,20^. The altered expression of miR-21 has been reported in the circulating exosomes of CRC patients by using miRNA microarray analysis^22^, but its potential role and underlying mechanism need to be further validated. Consistent with previous studies, miR-21-5p was overexpressed in CRC epithelium^19,27^. Interestingly, it was highly expressed in cancer-adjacent vessels, with an expression level that positively correlated with that in cancer epithelium. Moreover, we found that miR-21-5p can be transferred from CRC cells to endothelial cells via exosomes. Increased expression of miR-21-5p in recipient cells activates β-catenin signaling pathway by directly targeting KRIT1, contributing to angiogenesis and vascular permeability in CRC. Thus, these results suggest that exosomal miR-21-5p, secreted by CRC cells, is a crucial switch for cancer-induced angiogenesis and vascular permeability.

Exosomal miRNAs act as important mediators of intercellular communication, especially cancer-host crosstalk^7^. Cancer cells could induce chemotherapy resistance, microenvironment remodeling, pre-metastatic niche formation by releasing exosomes extracellularly to regulate stromal cells^6,7,15^, while exosomes secreted by stromal cells in turn alter cancer cells toward aggressive phenotypes^10,14^. For example, exosomal miR-221/222 secreted by tamoxifen-resistant breast cancer cells reportedly induced tamoxifen resistance in recipient ER-positive breast cancer cells^33^. Besides, exosomes derived from cancer-associated fibroblasts could elevate miR-92a-3p levels and promote metastasis and chemotherapy resistance in recipient CRC cells by inhibiting FBXW7 and MOAP1 (ref. ^14^). To conclude, these studies reveal that the delivery of exosomal miRNAs between cancer cells and stromal cells is involved in cancer progression. MiR-21 plays important roles in the proliferation, invasion, and metastasis of cancer epithelium and is associated with a poor prognosis of CRC^34^. Based on its stability and functionality, miR-21-5p has been studied a lot in CRC, while there is little research on miR-21-3p^19,21^. Herein, compared with corresponding controls, miR-21-5p was overexpressed in CRC epithelium and cancer-adjacent vessels. Interstingly, miR-21-5p expression levels in cancer epithelium and cancer-adjacent vessels were positively correlated. Even more, miR-21-5p expression levels in cancer epithelium positively correlated with micorvessels density. The above results suggested that miR-21-5p could be a potential linkage between cancer epithelium and cancer-adjacent vessels. Afterwards, treatment with SW480/miR-21-5p exosomes increased miR-21-5p expression, but not pri-miR-21, in recipient HUVECs, indicating that it was the exosomes-mediated transfer of mature miR-21-5p, but not other molecules, that resulted in up-regulation of miR-21-5p in HUVECs. Our results reveal that CRC-secreted miR-21-5p can be transferred to endothelial cells via exosomes.

Angiogenesis and vascular permeability are essential in facilitating tumor growth and metastases^12,13,35^. Recently, miR-21 has been reported to be up-regulated in cancer cells and induce angiogenesis by elevating levels of proangiogenic factors, such as vascular endothelial growth factor (VEGF), hypoxia-inducible factor-1α (HIF-1α), and angiopoietin 1 (ANG-1)^36,37^. Moreover, exosomes derived from adipose-derived stem cells overexpressing miR-21 enhanced the capacity of tube formation in recipient HUVECs^24^. Only four papers have reported miR-21’s roles in vascular permeability. MiR-21 reportedly disrupted the barrier function of the vascular endothelium and increased vascular permeability in Sprague Dawley rats exposed to Particulate Matter 2.5 (ref. ^38^). Although several studies have verified the essential roles of miR-21 in angiogenesis and vascular permeability^24,37,38^, the mechanisms through which CRC cells induce angiogenesis and vascular permeability remain unclear. Herein, we firstly extracted exosomes of CRC cells stably expressing or repressing miR-21-5p and detected the corresponding miR-21-5p expression levels. Afterwards, we incubated these exosomes with HUVECs and performed gain-of-function and loss-of-function assays to investigate the effect of exosomal miR-21-5p in endothelial cells. We found that the exosomal miR-21-5p released by CRC cells could promote proliferation and migration of HUVECs, and enhanced the capacity of tube formation and vessels leakiness in vitro. This finding has yielded a contradiction to the recent studies on the protective effect of miR-21 on vascular permeability in traumatic brain injury^39^ or renal delayed ischemic preconditioning^40^, which may be due to tissue heterogeneity or a loop regulation of miR-21. In addition, mice bearing miR-21-5p-overexpressed tumors induced rapid growth of new vessels in primary and metastatic tumors in the orthotopic xenografts model. Exosomal miR-21-5p facilitated vessels leakiness in vivo, as shown by more CRC cells were leaked into lung tissues to form colonization in tail vein metastatic mouse model. Our results suggest that exosomal miR-21-5p is required for cancer-induced angiogenesis and vascular permeability and can be a promising target for anti-angiogenesis-based CRC therapies.

To explore the underlying mechanisms of miR-21-5p in endothelial cells, we performed a bioinformatics search for potential target genes of miR-21-5p and focused on KRIT1. Only one paper has reported that miR-21 promotes cell growth on MDAMB231 (a breast cancer cell line) and MC-1 (a melanoma cell line) by directly targeting KRIT1 (ref. ^25^). KRIT1 is mainly reported in Cerebral Cavernous Malformation (CCM), a disease characterized by abnormally enlarged and leaky capillaries in the blood-brain barrier^41^. KRIT1, a component of the CCM signaling pathway, plays critical roles in vessel formation and integrity^31,41^. Moreover, the altered expression of KRIT1 was also detected in the intestinal epithelium and correlated with intestinal epithelial barrier maintenance and regulation^42^. However, there has been no published data about the roles of KRIT1 in cancer-adjacent vessels. In the present study, we focused on the roles of miR-21-5p and KRIT1 in cancer-adjacent vessels, but not cancer cells, which differed from previous studies. Treatment with exosomal miR-21-5p in HUVECs suppressed its direct target KRIT1, and reintroduction of KRIT1 could reverse exosomal miR-21-5p-induced angiogenesis and vascular permeability in vitro. Across a matched collection of 30 human CRC tissues, we detected a significant negative relationship between miR-21-5p and KRIT1 expression levels in CRC-adjacent vessels. Previous studies have noted that β-catenin interacts directly with the cytoplasmic tail of the vascular endothelial (VE)-cadherin, which mediates adherens junctions of endothelial cells^31^. Loss of CCM1 results in the release of β-catenin from VE-cadherin, subsequent nuclear translocation, and transcriptional activation, ultimately leading to potential activation of angiogenesis and disruption of adherens junctions^31,43^. Therefore we investigated whether exosomal miR-21-5p regulates angiogenesis and vascular permeability through the β-catenin signaling pathway or not. The current study provided evidences that exosomal miR-21-5p contributed to nuclear translocalization of β-catenin, activation of TCF/LEF-dependent transcription, and up-regulation of downstream genes VEGFa and Ccnd1, and these effects were significantly inhibited by overexpression of KRIT1 in recipient HUVECs. Collectively, this evidence clearly validates that exosomal miR-21-5p induces angiogenesis and vascular permeability by targeting KRIT1 and β-catenin/TCF activity in CRC.

Finally, we explored the clinical values of exosomal miR-21-5p in the progression of CRC. MiRNAs present extracellularly either through binding to protein or lipid carriers or as an important component of exosomes^16^. Exosomal miRNAs have been discovered to serve as reliable diagnostic biomarkers for cancer based on their stability with highly reproducible detection^44^. Exosomal miR-92a-3p was highly expressed in the serum and could be used as an early biomarker for metastasis and chemotherapy resistance in CRC patients^14^. In addition, exosomes have been proved to be an attractive and potentially effective tool of drug delivery in cancer therapy on account of their unique biocompatibility, high stability, preferred tumor homing and adjustable targeting efficiency^45^. Herein, exosomes released by CRC patients were rich in miR-21-5p, which distinguished CRC patients from healthy donors. Moreover, a significant decrease of miR-21-5p expression was detected in circulating exosomes of CRC patients who underwent surgical resection of the tumor. Therefore, quantitative blood test for exosomal miR-21-5p level can be considered as a potential biomarker for CRC.

**Fig. 6:**
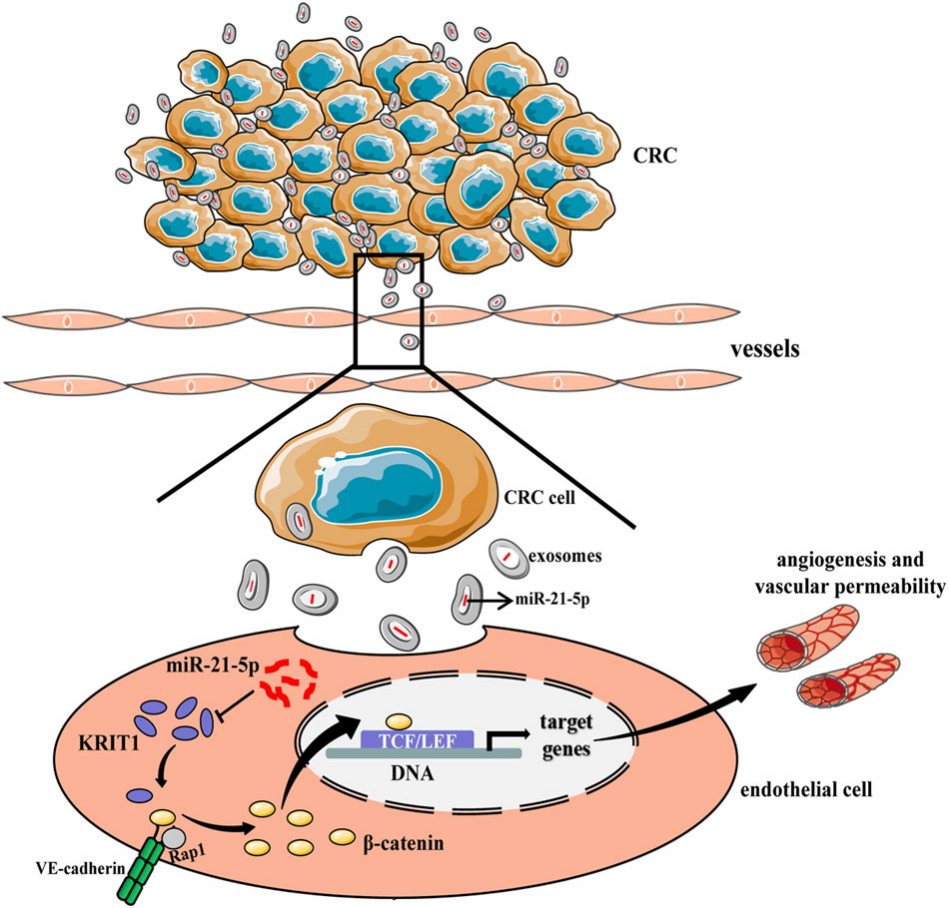
Schematic diagram of the role of exosomal miR-21-5p secreted by CRC in angiogenesis and vascular permeability. Endothelial cells uptake exosomes secreted by CRC cells, leading to an increase of miR-21-5p expression in recipient cells. Then miR-21-5p directly suppresses KRIT1 expression in recipient endothelial cells, subsequently releases β-catenin from VE-cadherin (a protein mediates adherens junctions of endothelial cells) to nuclear, following by TCF/LEF dependent transcription, consequently induces angiogenesis and vascular permeability in CRC.

In summary, our study shed light on poorly understood molecular mechanisms of exosomal miR-21-5p in the progression of CRC. We identified miR-21-5p was highly expressed in cancer-adjacent vessels, with an expression level that positively correlated with that in CRC epithelium. Notably, the incubation of exosomal miR-21-5p derived from CRC cells increased miR-21-5p expression in recipient HUVECs, subsequent suppressed KRIT1 and actived the β-catenin signaling pathway, consequently induced angiogenesis and vascular permeability in CRC (Fig. 6). Moreover, a negative relationship between miR-21-5p and KRIT1 expression levels was observed in cancer-adjacent vessels. To conclude, we propose exosomal miR-21-5p may be used as a useful therapeutic target to intervene angiogenesis and vascular permeability in CRC.

## Supplementary information

Supplementary Table 1

Supplementary figure legend

Supplementary figure 1

Supplementary figure 2

Supplementary figure 3
