# Supplementary material for: Cancer-secreted exosomal miR-21-5p induces angiogenesis and vascular permeability by targeting KRIT1
Source: Cell Death Dis. 2021 Jun 4;12(6):576. doi: 10.1038/s41419-021-03803-8 (PMC8178321; doi:10.1038/s41419-021-03803-8)
Supplement: Supplementary file 1 — Supplementary Table 1 [file 41419_2021_3803_MOESM1_ESM.doc]

**Supplementary Table 1: Primer sequences for Quantitative Real-Time PCR.**

| **Gene** | **Primers** | **Sequence (5’-3’)** |
| --- | --- | --- |
| KRIT1 | Forward | ATGCGAGTCTGTAGTGAATCCA |
|  | Reverse | TGTGCATGACGTTCATCTAACC |
| VEGFa | Forward | AGGGCAGAATCATCACGAAGT |
|  | Reverse | AGGGTCTCGATTGGATGGCA |
| CCND1 | Forward | CAATGACCCCGCACGATTTC |
|  | Reverse | CATGGAGGGCGGATTGGAA |
| GAPDH | Forward | TGTGGGCATCAATGGATTTGG |
|  | Reverse | ACACCATGTATTCCGGGTCAAT |
