# Supplementary material for: Cancer-secreted exosomal miR-21-5p induces angiogenesis and vascular permeability by targeting KRIT1
Source: Cell Death Dis. 2021 Jun 4;12(6):576. doi: 10.1038/s41419-021-03803-8 (PMC8178321; doi:10.1038/s41419-021-03803-8)
Supplement: Supplementary file 2 — Supplementary figure legend [file 41419_2021_3803_MOESM2_ESM.doc]

**Supplementary Figure 1** **CRC-secreted miR-21-5p is transferred to endothelial cells via exosomes** (A) Endogenous expression of miR-21-5p in 6 CRC cell lines by qRT-PCR. Mean ± SEM are provided (n = 3). (B) Expression of miR-21-5p in CRC cells transduced lentiviral vector expressing miR-21-5p or repressing miR-21-5p by qRT-PCR. The relative expression levels of miR-21-5p in SW480/mock, SW620/NC or SW480/NC cells were normalized to 1. Mean ± SEM are provided (n = 3). (C) Expression of miR-21-5p in exosomes derived from SW480/mock, SW480/miR-21-5p, SW620/NC, SW620/zip-miR-21-5p, SW480/NC or SW480/zip-miR-21-5p by qRT-PCR. The relative expression levels of miR-21-5p in SW480/mock exosomes, SW620/NC exosomes or SW480/NC exosomes were normalized to 1. Mean ± SEM are provided (n = 3). (D) The size distribution profile of exosomes derived from SW480 or SW620. (E) Expression of miR-21-5p and pri-miR-21 in HUVECs incubated with exosomes derived from SW480/NC or SW480/zip-miR-21-5p by qRT-PCR. Mean ± SEM are provided (n = 3). **P* < 0.05 ,***P* < 0.01, ****P* < 0.001. NS represents no significant difference.

**Supplementary Figure 2** **Exosomal miR-21-5p induces angiogenesis and vascular permeability in vitro and in vivo** (A) Effect of SW480/NC exosomes and SW480/zip-miR-21-5p exosomes on the proliferation of HUVECs. Mean ± SEM are provided (n = 3). (B) Effect of SW480/NC exosomes and SW480/zip-miR-21-5p exosomes on the migration of HUVECs by Boyden chamber. Scale bars represent 50µm. Mean ± SEM are provided (n = 3). (C) The number of GFP+ SW480 invaded through the HUVEC monolayers pre-treated with SW480/NC exosomes or SW480/zip-miR-21-5p exosomes. Scale bars represent 50µm. Mean ± SEM are provided (n = 3 ). (D) The volume of intestinal primary tumors (Intestine-pri), intestinal and hepatic metastatic tumors (Intestine-met and liver-met) after SW480/mock or SW480/miR-21-5p cells were injected in the cecal mesentery of nude mice for two months (n = 5 mice per group). The diameter of primary and metastatic tumors per mouse was measured under the microscope. Mean ± SEM are provided. (E) Effect of SW480/mock exosomes, SW480/miR-21-5p exosomes, SW620/NC exosomes and SW620/zip-miR-21-5p exosomes on vascular outgrowth by CAM assay. Mean ± SEM are provided (n = 3). **P* < 0.05 ,***P* < 0.01, ****P* < 0.001. NS represents no significant difference.

**Supplementary Figure 3** **Exosomal miR-21-5p induces angiogenesis and vascular permeability via regulating KRIT1 and β-catenin signaling pathway** (A) Luciferase activity of human wild-type 3′UTR-KRIT1-luc construct after transfection of miR-21-5p mimics or miR-21-5p inhibitor in HEK293A and HUVECs. Mean ± SEM are provided (n = 3). (B) Expression of KRIT1 in HUVECs treated with KRIT1 plasmid (lacking 3′UTR), siKRIT1-1, siKRIT1-2 or siKRIT1-3 by Western blot. Expression levels were normalized to β-actin. (C) The sequences of the conserved binding sites of miR-21-5p in the human and mouse KRIT1 3’UTR. The data was provided by TargetScan 7.0. (D) Luciferase activity of mouse wild-type 3′UTR-KRIT1-luc construct after transfection of miR-21-5p mimics or miR-21-5p inhibitor in HEK293A. Mean ± SEM are provided (n = 3). (E) Expression of KRIT1 in vessels (indicated by arrows) adjacent to primary CRC and matched normal mucosa after SW480/mock or SW480/miR-21-5p cells were injected in the cecal mesentery of nude mice for two months (n = 5). Scale bars represent 50µm. (F) Expression of VEGFα and CCND1 in HUVECs treated with SW480/mock exosomes, SW480/miR-21-5p exosomes, SW480/miR-21-5p exosomes+KRIT1, SW620/NC exosomes, SW620/zip-miR-21-5p exosomes or SW620/zip-miR-21-5p exosomes+siKRIT1 by qRT-PCR. Mean ± SEM are provided (n = 3). (G) Expression of KRIT1 in vessels (indicated by arrows) adjacent to liver metastasis of CRC and matched adjacent liver by IHC. Scale bars represent 50µm. **P* < 0.05 ,***P* < 0.01, ****P* < 0.001.
