# Supplementary figures and images for: Cancer-secreted exosomal miR-21-5p induces angiogenesis and vascular permeability by targeting KRIT1
Source: Cell Death Dis. 2021 Jun 4;12(6):576. doi: 10.1038/s41419-021-03803-8 (PMC8178321; doi:10.1038/s41419-021-03803-8)

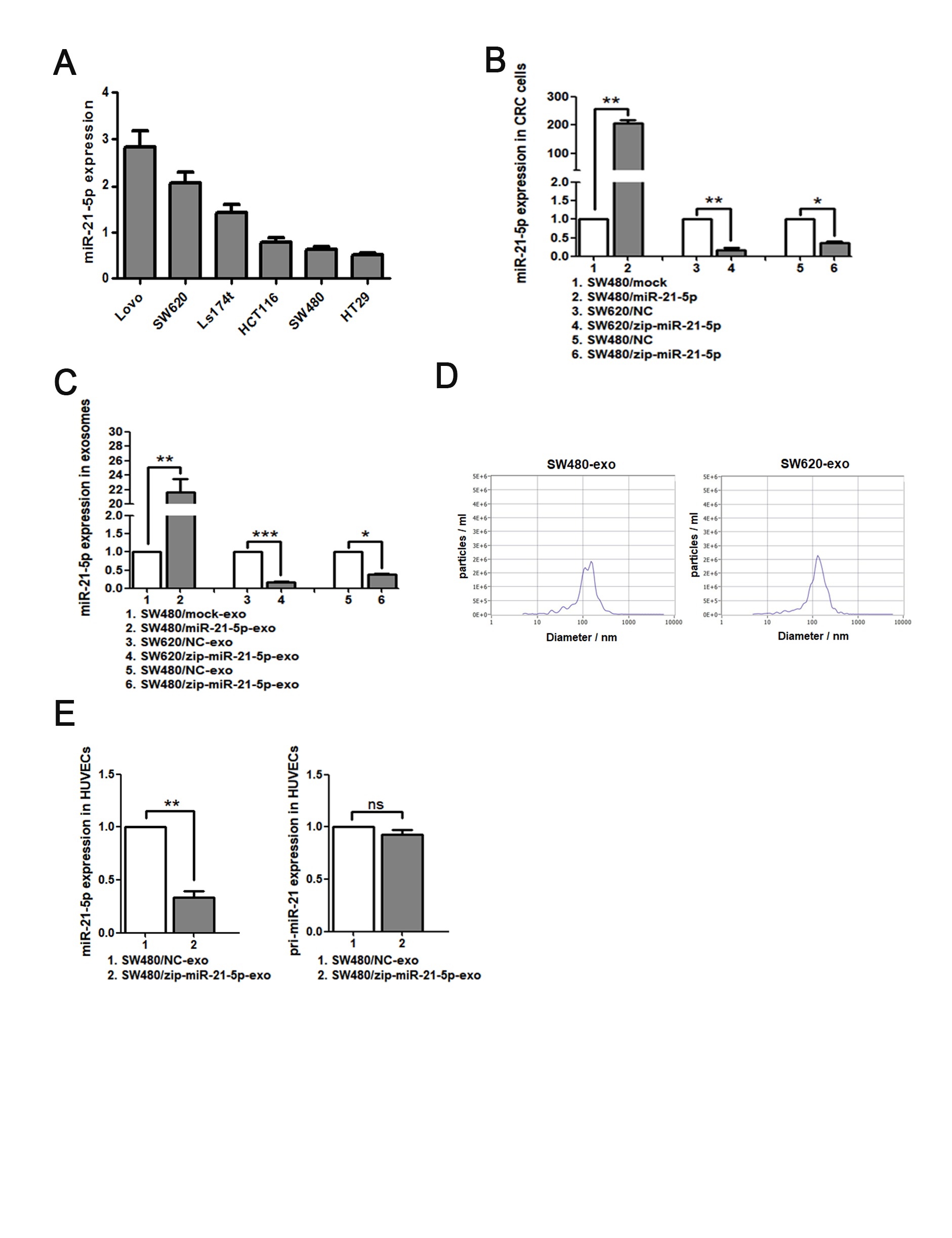

Supplement: Supplementary file 3 — Supplementary figure 1 [file 41419_2021_3803_MOESM3_ESM.tif]

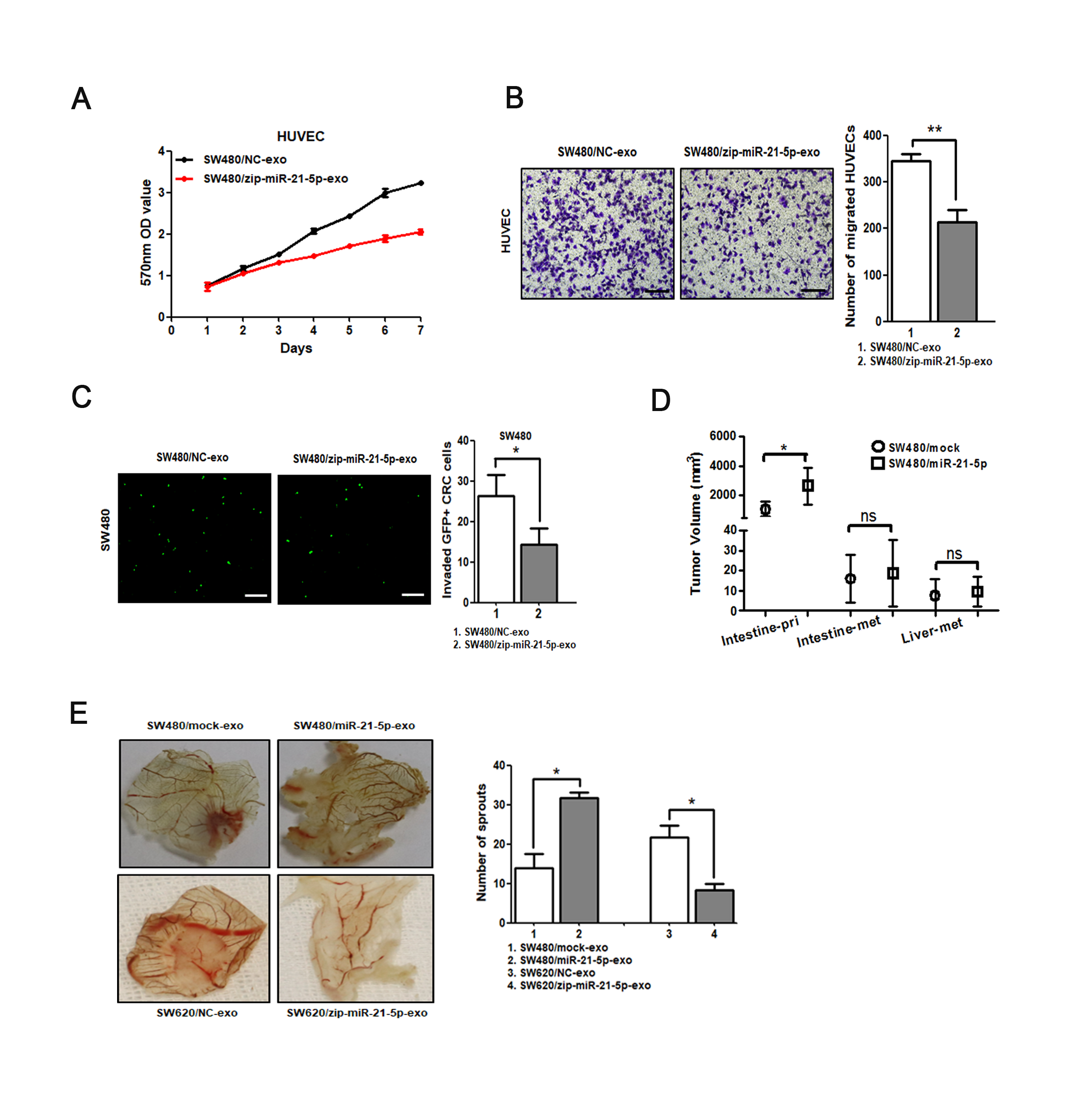

Supplement: Supplementary file 4 — Supplementary figure 2 [file 41419_2021_3803_MOESM4_ESM.tif]

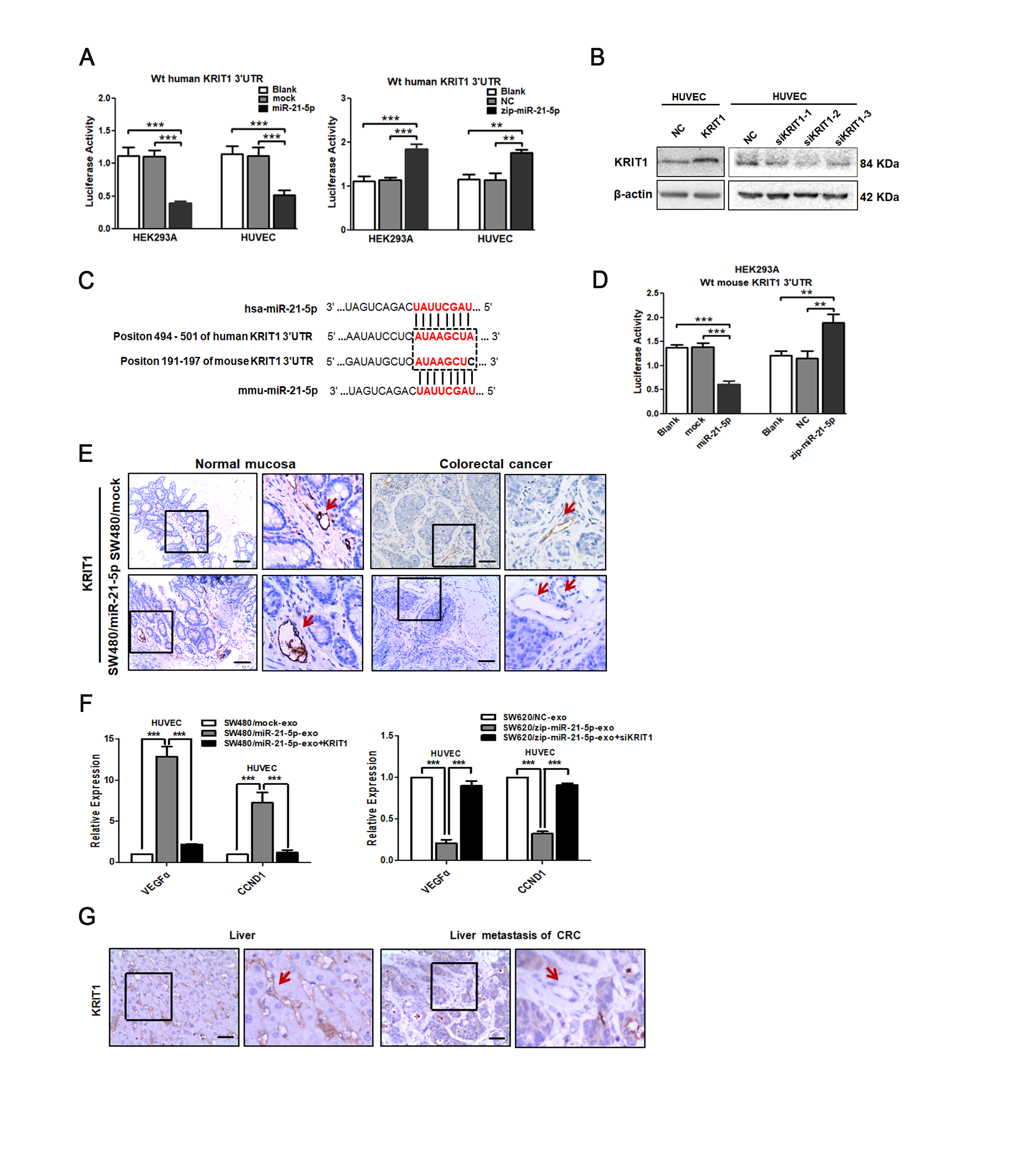

Supplement: Supplementary file 5 — Supplementary figure 3 [file 41419_2021_3803_MOESM5_ESM.tif]
